# Iron therapy for renal anemia: how much needed, how much harmful?

**DOI:** 10.1007/s00467-006-0405-y

**Published:** 2007-04-01

**Authors:** Walter H. Hörl

**Affiliations:** Division of Nephrology and Dialysis, Department of Medicine III, Medical University of Vienna, Währinger Gürtel 18-20, 1090 Vienna, Austria

**Keywords:** Iron supplementation, Iron status, Inflammation, Infection, Erythropoiesis-stimulating agents

## Abstract

Iron deficiency is the most common cause of hyporesponsiveness to erythropoiesis-stimulating agents (ESAs) in end-stage renal disease (ESRD) patients. Iron deficiency can easily be corrected by intravenous iron administration, which is more effective than oral iron supplementation, at least in adult patients with chronic kidney disease (CKD). Iron status can be monitored by different parameters such as ferritin, transferrin saturation, percentage of hypochromic red blood cells, and/or the reticulocyte hemoglobin content, but an increased erythropoietic response to iron supplementation is the most widely accepted reference standard of iron-deficient erythropoiesis. Parenteral iron therapy is not without acute and chronic adverse events. While provocative animal and in vitro studies suggest induction of inflammation, oxidative stress, and kidney damage by available parenteral iron preparations, several recent clinical studies showed the opposite effects as long as intravenous iron was adequately dosed. Thus, within the recommended international guidelines, parenteral iron administration is safe. Intravenous iron therapy should be withheld during acute infection but not during inflammation. The integration of ESA and intravenous iron therapy into anemia management allowed attainment of target hemoglobin values in the majority of pediatric and adult CKD and ESRD patients.

## Introduction

Iron-restricted erythropoiesis is a common clinical condition in patients with chronic kidney disease (CKD). The causes underlying this pathology and the subsequent contribution of absolute or functional iron deficiency to renal anemia include:

- Inadequate intake of dietary iron
- Blood loss during the extracorporeal procedure in hemodialysis patients
- Blood loss from the gastrointestinal tract (bleeding)
- (Too) frequent diagnostic blood tests
- Inadequate intestinal iron absorption and inhibition of iron release from macrophages (anemia of chronic disease)
- Increased iron requirements during therapy with erythropoiesis-stimulating agents (ESAs).

In iron deficiency without anemia, reduction in iron storage is not sufficiently large enough to decrease the hemoglobin level. In CKD patients with absolute iron-deficient anemia, however, iron deficit is so severe that it aggravates renal anemia. Iron supplementation is mandatory in the majority of patients with end-stage renal disease (ESRD), particularly in those receiving ESA therapy.

## Evaluation of iron status

In patients with normal kidney function, absolute iron deficiency is characterized by low serum ferritin concentration (<30 μg/l). The ferritin cut-off level for absolute iron deficiency in CKD patients is 100 μg/l [1] by the experience that chronic inflammation increases serum ferritin levels approximately three-fold. The Kidney Disease Outcomes Quality Initiative (K/DOQI) guidelines recommend serum ferritin levels >200 μg/l for the adult hemodialysis patient population [2]. The European Best Practice Guidelines define the optimal range for serum ferritin as 200–500 μg/l in adult patients with ESRD [1]. A normal ferritin level (≥100 μg/l) cannot exclude iron deficiency in uremic children [3, 4], but a serum ferritin <60 μg/l is a specific predictor of its presence [5]. An upper ferritin level of 500 μg/l is recommended for adults and children with CKD [2]. Serum ferritin is an indicator of storage iron. Iron deficiency is accompanied by reductions in serum iron concentration and transferrin saturation (TSAT) and by elevations in red cell distribution width, free erythrocyte protoporphyrin concentration, total serum iron binding capacity (TIBC), and circulating transferrin receptor [6]. Serum soluble transferrin receptor, however, reflects ongoing erythropoiesis but not iron availability in ESA-treated chronic dialysis patients [7]. Typically, TSAT (the ratio of serum iron to TIBC) is 15% or less (normal 16–40%) with iron deficiency, but TSAT also decreases in the presence of acute and chronic inflammation (functional iron deficiency). TSAT is raised with bone marrow dysfunction due to alcohol, cancer chemotherapy, or a megaloblastic process. TSAT is also affected by diurnal variations, being higher in the morning and lower in the evening [8]. Even a TSAT > 20% or a serum ferritin level > 200 μg/l does not exclude iron deficiency in ESRD patients. In a study by Chuang et al. [9], 17% of iron-deficient hemodialysis patients had serum ferritin levels greater 300 μg/l. Clinically, functional iron deficiency is confirmed by the erythropoietic response to a course of parenteral iron and is excluded by the failure of erythroid response to intravenous iron administration [10].

Erythrocyte and reticulocyte indices, such as the percentage of hypochromic red blood cells and the reticulocyte hemoglobin content (CHr) provide direct insight into bone marrow iron supply and utilization. Determination of the percentage of hypochromic red blood cells, i.e., those with a cellular hemoglobin concentration <28 g/dl, provides important information on functional iron deficiency in ESA-treated dialysis patients [11]. Tessitore et al. [12] found that hypochromic red blood cells >6% are the best marker to identify adult ESRD patients who will have the best response to intravenous iron. CHr has been proposed as a surrogate marker of iron status and as an early predictor of response to iron therapy in adult dialysis patients [13, 14]. Combined use of CHr and high-fluorescence reticulocyte count predicts with a very high sensitivity and specificity the response to intravenous iron in adult dialysis patients [8]. There are, however, only few studies in the pediatric renal literature on the use of CHr [15, 16]. In children with ESRD, an increase from baseline CHr levels was observed in response to oral and intravenous iron, but cut-off values for the use of CHr in the pediatric CKD population are not clear. This measure has proven to be of value with adult ESRD patients.

Detection of both absolute and functional iron deficiency is important because iron deficiency is the most common cause of hyporesponsiveness to ESAs. In clinical practice, an increased erythropoietic response to iron supplementation is the most widely accepted reference standard of iron-deficient erythropoiesis. For pharmacological therapy of iron deficiency, both oral and parenteral iron preparations are available. Intravenous iron is more effective than oral iron supplementation, at least in CKD patients. Iron is not only a prerequisite for effective erythropoiesis but also an essential element in all living cells. Elemental iron serves as a component of oxygen-carrying molecules and as a cofactor for enzymatic processes. Its redox potential, however, limits the quantity of iron that can be safely harbored within an individual.

## Oral iron therapy

Oral iron is best absorbed if given without food. Side-effects of oral iron therapy include constipation, diarrhea, nausea, and abdominal pain. In the treatment of iron deficiency with ferrous sulphate, the usual adult dose is one 300 mg tablet (containing 60 mg elemental iron) three to four times daily. The pediatric dose is 2–6 mg/kg per day of elemental iron in 2–3 divided doses [17, 18]. Intestinal iron absorption is enhanced in patients with iron deficiency and declines with the correction of iron deficiency and reaccumulation of iron stores. If side-effects limit compliance, the medication can be administered with food, or the dose can be reduced. One 500-mg ferrous sulphate dose nightly at bedtime may be an effective therapy in adults [19].

Uremia is a chronic inflammatory state [20, 21]. Even in the absence of overt infection or inflammation, many ESRD patients show increased levels of acute-phase proteins, such as C-reactive protein (CRP), ferritin, fibrinogen, and/or interleukin-6 (IL-6), associated with low serum albumin levels [22]. The interaction of proinflammatory cytokines with hepcidin in mediating functional iron deficiency may explain why CKD patients have high ferritin levels, poor intestinal iron absorption, and disturbed iron release from the reticuloendothelial system [23].

In the duodenum and proximal jejunum, the nonheme dietary Fe^3+^ is reduced to Fe^2+^ by the cytochrome b-like ferrireductase Dcytb. Fe^2+^ is gathered from the lumen of the intestine and crosses the apical enterocyte brush border membrane through the divalent metal transporter-1 (DMT1). The expression of both Dcytb and DMT1 is strongly affected by the iron concentration within the enterocyte. Circulating levels of hepcidin negatively regulate intestinal iron absorption by the enterocyte DMT1. Hypoxia, anemia, iron deficiency, and/or stimulated erythropoiesis strongly down-regulate hepatic hepcidin release, allowing intestinal iron absorption, while iron overload or inflammation/infection stimulates hepcidin production, resulting in inhibition of intestinal iron absorption. Hepcidin controls the whole-body iron content. It also inhibits the release of iron by the iron exporter ferroportin (iron-regulated transporter-1) located along the entire basolateral membrane of enterocytes and also in the intracellular vesicular compartment of tissue macrophages. Hepcidin is primarily produced in the liver in response to acute-phase reactions. Any further expression depends on the degree of hepatic iron storage (for review, see [24]). Thus, the inflammatory state associated with uremia and less uremia per se is predominantly responsible for poor intestinal iron absorption in ESRD patients. In iron-depleted peritoneal dialysis patients with normal CRP values, high-dose oral iron is well absorbed [25].

The European Pediatric Peritoneal Dialysis Working Group recommended that anemia treatment should aim for a target hemoglobin concentration of at least 11 g/dl accomplished by administration of ESA and iron. Oral iron should be preferred in pediatric peritoneal dialysis patients [18]. The majority of pediatric hemodialysis patients are also supplemented with oral iron. The 2001 North American Pediatric Renal Transplant Cooperative Study (NAPRTCS) annual report showed that 84% of pediatric peritoneal dialysis and 72% of pediatric hemodialysis patients at 12 months of dialysis were receiving oral iron therapy [26].

## Intravenous iron therapy

Since oral iron therapy is often not sufficient in ESRD patients, parenteral administration of iron is necessary to optimally care for these patients. Intravenous iron can be given safely to CKD patients [27–34] as long as the therapy is performed according to international recommendations and guidelines [1, 2]. This therapy is unequivocally superior to oral iron supplementation [35]. All forms of intravenous iron may be associated with acute adverse events [1, 2]. Potential risk factors associated with intravenous iron therapy include acute allergic reactions such as rash, dyspnoea, wheezing, or even anaphylaxis, as well as long-term complications caused by the generation of powerful oxidant species, initiation and propagation of lipid peroxidation, endothelial dysfunction, propagation of vascular smooth muscle cell proliferation, and/or inhibition of cellular host defense. Allergy is believed to relate to dextran moiety. Iron dextran therapy is associated with a higher risk for serious type I reactions compared with newer intravenous iron products. Iron sucrose carries the lowest risk for hypersensitivity reactions [36]. In our clinical experience with more than 100,000 intravenous injections of iron sucrose and ferric gluconate within the last 15 years, we detected no significant differences in efficacy or adverse events between both intravenous iron preparations. Serious reactions to iron dextran are unpredictable and possibly life threatening. Labile- or free-iron reactions are more frequent with nondextran forms [37]. Recommended doses of iron sucrose or ferric gluconate appear safe, at least in adult CKD patients [32, 34, 38]. Parenteral therapy with iron sucrose or ferric gluconate is also safe and effective in the management of anemia in adult hemodialysis patients sensitive to iron dextran [29, 39]. Iron sucrose safety data are sparse in the pediatric CKD literature [2]. It should be considered that the iron load administered intravenously to CKD patients based on international recommendations is more than ten times less than the iron load by repeated blood transfusions at times when no ESA therapy was available for ESRD patients.

Iron deficiency in CKD patients develops primarily during the correction of renal anemia by ESA treatment. Approximately 150 mg of iron is necessary for an increase of 1 g/dl in hemoglobin level. In adult hemodialysis patients, annual blood losses up to 4 l of blood, equivalent to 2 g iron, should be considered [40]. Thus, intravenous iron prevents iron-restricted erythropoiesis during ESA therapy. Parenteral treatment strategies depend on the availability of iron products in respective countries. Hemodialysis patients should receive at least one dose of intravenous iron every 2 weeks [1]. Careful monitoring of iron status is mandatory in order to avoid iron overload. In patients with anemia of chronic disease (and inflammation), a major part of iron administered intravenously is transported into the reticuloendothelial system, where it is not readily available for erythropoiesis [41].

Intravenous iron therapy is underused in pediatric ESRD patients. Chavers et al. [42] compared anemia prevalence in US Medicare pediatric and adult dialysis patients treated with ESAs from 1996 to 2000. Prevalence of anemia (defined as hemoglobin values less than 11 g/dl) was found in pediatric and adult hemodialysis patients during 54.1% versus 39.8% patient years as well as in pediatric and adult peritoneal dialysis patients during 69.5% versus 55.1% patient years, respectively. The percentage of patient years with intravenous iron was low, especially for pediatric peritoneal dialysis patients: 33.9% (age group 0–4 years) and 71% (age group 5–19 years) versus 0.3% and 19.4% among pediatric hemodialysis patients in these age categories, respectively. Among pediatric hemodialysis and peritoneal dialysis patients, intravenous iron was not administered among 34% and 85% of patient years [42]. Data obtained from the US Centers for Medicare and Medicare Services on hemodialysis patients in an age range between 12 and <18 years indicate that 37% of these patients are anemic, defined as hemoglobin <11 g/dl. Dialyzing <6 months, a low serum albumin, and a mean TSAT <20% were identified as predictors of anemia in these children. Despite the prescription of iron supplements in almost all pediatric patients, there was evidence for low TSAT and/or low ferritin in many children. In this study, approximately 60% of all children received intravenous iron therapy [43].

An international multicenter study investigated the safety and efficacy of two dosing regimens (1.5 mg/kg or 3 mg/kg) of ferric gluconate during eight consecutive hemodialysis sessions in iron-deficient pediatric hemodialysis patients receiving concomitant ESA therapy. Efficacy and safety profiles were comparable, with no unexpected adverse events with either dose [16]. Initial recommended ferric gluconate therapy is 1.5 mg/kg for eight doses for iron-deficient pediatric hemodialysis patients and 1 mg/kg per week for iron-replete pediatric hemodialysis patients, with subsequent dose adjustments made according to TSAT and/or ferritin levels [16, 44]. In children, iron sucrose in a high dose (5 mg/kg) should be given over 90 min and in a low dose (up to 2 mg/kg) over 3 min [45]. An other recommendation for pediatric patients is to inject 6 mg iron/kg per month during iron deficiency, with subsequent dose adjustments according to serum ferritin [46]. Nonrandomized intravenous iron (1–4 mg/kg per week) trials in children on hemodialysis and nondialyzed or transplanted children showed an increase in hemoglobin or hematocrit and a decrease in ESA requirements between 5% and 62% per week or per dose of ESA [3, 4, 47, 48]. De Palo et al. [49] reported an excessive increase in hemoglobin with severe hypertension in children on maintenance hemodialysis after the first month of darbepoetin alpha therapy combined with intravenous ferric gluconate in a very high dose of 10–20 mg/kg per week. The patients had already been on erythropoietin therapy for at least 6 months with adequate iron status (serum ferritin 220 ± 105 μg/l; TSAT 24.2 ± 11.5%). The complications observed in this study are not surprising, as such a high-dosed intravenous iron therapy is not justified, either in adult or in pediatric dialysis patients with “adequate iron status”. The current practice of intravenous iron therapy in pediatric hemodialysis patients is often performed on extrapolation from adult data and not based on data obtained from prospective multicenter trials performed in children [50].

Even if exposure to intravenous iron may lead to oxidative stress, renal injury, infection, and/or cardiovascular disease, the magnitude of these complications is not really clear. The overall risk–benefit ratio favors the use of intravenous iron in CKD patients in order to optimize erythropoiesis and prevent iron deficiency [51]. Intravenous iron therapy is still underutilized in the adult hemodialysis population [52], but its use increased from 1997 to 2002. Ferric gluconate and iron sucrose have become the predominant form of intravenous iron therapy [53].

## Iron and inflammation/infection

Intravenous iron therapy may adversely impact CKD patients via a potentiation of systemic inflammation. In animals, even a single ultra-high-dosed intravenous injection of available iron preparations (2 mg iron for mice with a body weight of 25–35 g, corresponding to 5,000 mg iron for a 75-kg adult patient) does not independently raise plasma levels of tumour necrosis factor-α (TNF-α). Systemic inflammation experimentally induced by intraperitoneal endotoxin injection (2 or 10 mg/kg in mice) resulted in a dramatic increase in plasma TNF-α levels. Interestingly, 2 h following concomitant injection of endotoxin and ferric gluconate (2 mg) or iron dextran (2 mg), a decrease of plasma TNF-α levels was observed. In contrast, combined endotoxin and iron sucrose injection resulted in a further increase in plasma TNF-α compared with endotoxin alone [54]. However, a 75-kg CKD patient will neither receive 5,000–25,000 mg endotoxin intraperitoneally nor concomitant 5,000 mg iron sucrose injection intravenously in order to demonstrate that under these artificial conditions, TNF-α mRNA and TNF-α release are stimulated. It is therefore of particular importance that relevant clinical studies demonstrated that intravenous iron sucrose therapy within recommended doses may even display anti-inflammatory effects [55, 56].

Intravenous iron sucrose therapy affects positively circulating cytokine levels in hemodialysis patients: IL-4 levels increase, while TNF-α levels decrease [55]. There is a direct correlation between IL-4 and TSAT but an inverse correlation between TNF-α and TSAT. Hemoglobin levels increase with an increase of IL-4 and a decrease of TNF-α, while ESA dose decreases with an increase of IL-4 and a decrease of TNF-α [55]. In other words, adequately dosed intravenous iron therapy in hemodialysis patients results in down-regulation of proinflammatory immune effector pathways and stimulation of the expression of the anti-inflammatory cytokine IL-4. By these mechanisms, in addition to its well-known stimulatory effects on erythropoiesis, iron therapy contributes to an increase in hemoglobin levels and to a decrease in the need of ESAs. The anti-inflammatory properties of intravenous iron therapy have also been demonstrated in patients with rheumatoid arthritis [56].

In contrast, iron-mediated weakening of the Th-1 immune effector function (estimated by lowered TNF-α production) with a subsequent strengthening of Th-2-mediated immune effector function (estimated by increased IL-4 production) is an unfavorable condition for ESRD patients in the case of an acute infection or malignant disease [55]. Moreover, intravenous administration of iron increases the availability of this essential nutrient for microorganisms [57] associated with an increased incidence of infectious complications in ESRD patients. Teehan et al. [58] followed 132 hemodialysis patients for up to 1 year after the initiation of intravenous iron therapy for the outcome of bacteremia. Iron-replete patients (those with a TSAT value ≥ 20% and a ferritin level ≥ 100 ng/ml) had a significantly higher risk of bacteremia (hazard ratio 2.3 in the univariate analysis and 2.5 in the multivariate analysis) compared with adult hemodialysis patients who were not iron replete [58]. Inhibition of intracellular killing of bacteria by polymorphonuclear leukocytes (PMNL) due to iron sucrose therapy in high-ferritin hemodialysis patients has been reported [59]. Peritoneal dialysis patients receiving high-dose intravenous iron sucrose also displayed short-term inhibition of bacterial killing by PMNL [60]. Finally, iron sucrose as well as ferric gluconate inhibit in vitro migration of PMNL through endothelial cells [61]. All these data suggest a risk for infectious complications, at least in patients overtreated with iron.

However, clinical studies on intravenous iron therapy in ESRD patients reported controversial results [62–64]. According to the published cohort study, among 32,566 hemodialysis patients, there was no association between iron administration and mortality. This study by Feldman and coworkers [64] supports intravenous administration of iron ≥1,000 mg over 6 months if needed to maintain target hemoglobin levels. This is, however, an adult and not a pediatric recommendation. Intravenous iron therapy should be withheld in the presence of acute infection until the infection has successfully been treated and resolved [65]. Intravenous iron is ineffective and may increase the virulence of bacterial and viral pathogens. On the other hand, ESRD patients with chronically infectious complications may develop absolute iron deficiency if iron supplementation is withheld over months. In such a situation, iron should be administered intravenously as soon as ferritin levels drop below 100 μg/l (personal opinion). ESA-stimulated erythropoiesis of chronically infected adult ESRD patients may benefit from low-dose intravenous iron supplementation (10–20 mg iron sucrose or ferric gluconate per hemodialysis session), even if serum ferritin is normal or slightly elevated. The level of serum ferritin at which ESRD patients are considered to be in an iron overload state is still not defined. Inflammatory states should not be considered indiciations to withhold the benefits of intravenous iron therapy in general [65]. However, a clinical problem is the diagnosis of chronic anemia associated with inflammation and true iron deficiency, as serum ferritin concentration increases rather than decreases.

## Iron and kidney function

Intravenous administration of 100 mg iron sucrose in CKD patients caused transient proteinuria and tubular damage [37], but ferric gluconate did not (125 mg infused over 1 h or 250 mg infused over 2 h) [66]. Induction of passive Heymann nephritis in rats resulted in a marked increase in nonheme iron content of kidney cortex and tubules, while a iron-deficient diet caused a significant reduction of nonheme iron level in glomeruli and also a significant reduction of proteinuria in these animals [67]. Pediatric thalassemia patients have a high prevalence of renal tubular abnormalities, probably caused by the anemia and increased oxidative stress induced by excess iron deposits. Significantly higher levels of urinary N-acetyl-beta-d-glucosaminidase, malondialdehyde, and beta-2-microglobulin were found in these children compared with normal children [68]. Under artificial experimental conditions (intravenous injection of 2 mg iron sucrose or ferric gluconate into mice with a body weight of 25–35 g), induction of monocyte chemoattractant protein-1 (MCP-1) in renal and extrarenal tissues has been observed. Since MCP-1 has profibrotic properties, implications for CKD progression in case of intravenous iron therapy has been suggested [69]. However, in a recent article, Mircescu et al. [70] reported that intravenous iron sucrose therapy (200 mg elemental iron per month for 12 months) resulted in an increase in hemoglobin from 9.7 ± 1.1 to 11.3 ± 2.5 g/dl in nondiabetic patients with CKD and a mean glomerular filtration rate (GFR) of 36.2 ± 5.2 ml/min per 1.73 m^2^, estimated by the formula of Cockcroft and Gault. The majority of these CKD patients had preexisting iron deficiency (mean ferritin 98.0 μg/l, range 24.8–139.0 μg/l). An important finding of this study was that GFR (final values at the end of the study 37.2 ± 0.9 ml/min per 1.73 m^2^) remained completely stable over a period of 12 months despite 2,400 mg of intravenous iron sucrose administration. The CKD patients had relatively high blood pressure (140 ± 32/82 ± 20 mmHg at baseline), which did not change throughout the investigation [70]. Agarwal [71] found that a single dose of 100 mg iron sucrose results in a transient increase of MCP-1 in plasma and urine of CKD patients. Those who believe that 100 mg iron sucrose administered to CKD patients may negatively affect kidney function should simply administer a lower intravenous iron dose, e.g., 50 mg iron sucrose intravenously.

## Iron and oxidative stress

Zager et al. [72] compared in vitro parenteral iron toxicity induced by three commercially available iron preparations (iron dextran, ferric gluconate, iron sucrose) using renal tubular cells and renal cortical homogenates. Each test agent induced massive and similar degrees of lipid peroxidation. Under the in vitro conditions used, iron sucrose caused markedly higher cell death than ferric gluconate, and ferric gluconate caused higher cell death than iron dextran. This relative toxicity profile was also observed in cultured aortic endothelial cells. Again, it should be stressed that the study of Mircescu et al. [70] demonstrated that intravenous iron sucrose therapy administered within international recommendations (none of the 58 CKD patients exceeded serum ferritin of 500 μg/l) does not cause a decline in kidney function in CKD patients over a period of 1 year.

Intravenous iron therapy may enhance symptoms of oxidative stress [73–75]. Drüeke et al. [75] demonstrated that advanced oxidation protein products (AOPPs) correlated with iron exposure and carotis artery intima thickness in dialysis patients. In hemodialysis patients, oxidative stress as a result of intravenous iron therapy caused serum albumin oxidation [76]. Ferric gluconate modifies fibrinogen and β_2_-microglobulin as a marker of oxidative stress in adult hemodialysis patients [77, 78]. Intravenous administration of 100 mg iron sucrose in CKD patients increased malondialdehyde as a marker of lipid peroxidation [37]. Hemodialysis patients with ferritin levels above 650 μg/l showed an enhanced oxidative burst in PMNL [59]. However, not all studies found evidence for enhanced oxidative stress caused by parenteral iron therapy in ESRD patients. Hemodialysis therapy per se was found to cause a significant increase in peroxide concentration. Interestingly, this rise in plasma total peroxides was not additionally influenced by concomitant intravenous injection of 100 mg iron sucrose [79]. These data confirm increased oxidative stress associated with hemodialysis [80]. Whether intravenous iron therapy results in an additional oxidative stress reaction needs to be further evaluated.

Increased blood levels of non-transferrin-bound iron (NTBI) and/or its redox-active part have been reported in adult ESRD patients receiving intravenous iron therapy [79, 81, 82]. Intravenous infusion of 300 mg iron sucrose in ESRD patients also caused peripheral vasodilation, which was confirmed by increased forearm blood flow. NTBI and redox-active iron were considered to be, at least in part, responsible for endothelial dysfunction observed in ESRD patients. However, an increase in NTBI and redox-active iron caused by intravenous iron sucrose infusion did not influence vascular reactivity to intra-arterial acetylcholine, glycerol-trinitrate, or L-N-mono-methyl-arginine (L-NMMA) [82].

## Vitamin C and iron

ESRD patients undergoing regular hemodialysis or hemodiafiltration may develop vitamin C deficiency [83]. Vitamin C deficiency may cause oxidative stress and vascular complications as well as impairment of intestinal iron absorption and iron mobilization from iron stress. Moretti et al. [84] measured iron absorption in young women from test meals fortified with isotopically labeled ferric pyrophosphate and ferrous sulfate. The addition of ascorbic acid at a molar ratio of 4:1 to iron increased iron absorption from ferric pyrophosphate to 5.8% and that from ferrous sulfate to 14.8%. In the fasting state, ferrous ascorbate is better absorbed than ferric hydroxide-polymaltose complex [85]. High-dose oral vitamin C may increase intestinal aluminium absorption [86]. Oxidative stress can cause hyporesponsiveness to ESA therapy in ESRD patients. Vitamin C may improve erythropoiesis through its antioxidative properties [87]. Intravenous ascorbic acid therapy facilitates iron release from inert deposits, resulting in a decrease of soluble transferrin receptor and an increase of TSAT [88]. In contrast, oral vitamin C supplementation (250 mg three times per week for 2 months) did not influence oxidative/antioxidative stress and inflammation markers in adult hemodialysis patients [89].

In adult hemodialysis patients on maintenance intravenous iron sucrose therapy, intravenous administration of 500 mg ascorbic acid three times a week for 6 months resulted in an increase of TSAT and hemoglobin in approximately 65% of the patients [90]. In contrast, neither oral nor intravenous ascorbic acid changed TSAT or hemoglobin levels in a study performed by Chan et al. [91]. Ascorbic acid increases the intracellular labile iron pool and iron mobilization to transferrin in human hepatoma HepG2 cells only in the presence of iron sucrose but not in the presence of iron dextran or ferric gluconate [92]. Several studies reported an increase in hemoglobin and/or a decrease in adult ESA dose during adjuvant ascorbic acid therapy three times per week in ESRD patients [93–99]. Measurements of plasma oxalate concentration are needed in ESRD patients supplemented with ascorbic acid [100]. Studies on vitamin C and iron in children with ESRD are not available so far.

## Iron therapy after kidney transplantation

Anemia is observed in 21–39.7% of adult renal transplant patients [101–104]. The prevalence may be even higher in pediatric transplant recipients: 84.3% of children were anemic in the first month after kidney transplantation, and prevalence of anemia was not below 64.2% between 6 months and 6 years after transplantation. Iron deficiency was identified in 27–56% of children between 1 and 60 months posttransplantation [105]. Fourteen pediatric and young adult renal transplant recipients received single iron gluconate infusions ranging from 1.9 to 6.4 mg/kg. The mean hemoglobin level increased significantly from 10.1 ± 1.6 to 11.4 ± 2.1 g/dl following ferric gluconate therapy. Adverse events were observed in three children [106]. A recent study by Morii and coworkers showed that oral coadministration of ferrous sulphate markedly decreased the absorption of mycophenolate mofetil in healthy Japanese subjects [107]. However, a randomized crossover trial failed to confirm this observation in European transplant patients receiving long-term mycophenolate mofetil therapy [108]. In line with this observation, an in vitro study showed that iron ions did not interact with mycophenolate mofetil [109].

## Conclusions

The integration of ESA and intravenous or oral iron therapy into standard anemia management resulted in target hemoglobin levels (as established by international guidelines) in the vast majority of ESRD patients [110]. Correction of renal anemia reduced morbidity and mortality as well as hospitalization in ESRD patients. It also improved quality of life, cognitive function, and physical activity. Using a balanced approach to iron supplementation within international recommendations allowed the attainment of benefits of intravenous iron therapy at storage iron levels far below those generally seen with transfusions in the pre-ESA era [110].
